# hAMSC Sheet Promotes Repair of Rabbit Osteochondral Defects

**DOI:** 10.1155/2022/3967722

**Published:** 2022-03-31

**Authors:** Gang Zou, Jun Zhang, Qifan Yang, Xiaoyan Wang, Pengpeng Sun

**Affiliations:** ^1^Department of Orthopaedic Surgery, Affiliated Hospital of Zunyi Medical University, China; ^2^Department of Orthopedics, The First Affiliated Hospital of Chongqing Medical University, Chongqing 400016, China; ^3^Department of Obstetrics and Gynecology, Affiliated Hospital of Zunyi Medical University, China

## Abstract

Osteochondral lesion is clinically common disease, which has been recognized as one of the contributing factors of significant morbidity. Although current treatments have achieved good outcomes, some undesirable complications and failures are not uncommon. Cell sheet technology (CST), an innovative technology to harvest seed cells and preserve abundant ECM, has been widely used in various tissue regeneration. For osteochondral lesion, many studies focus on using CST to repair osteochondral lesion and have achieved good outcomes. In the previous study, we have demonstrated that hAMSC sheet had a positive effect on osteochondral lesion. Therefore, this study is aimed at comparing the effect of noninduced hAMSC sheet with chondrogenically induced hAMSC sheet on osteochondral lesion and cartilage regeneration.

## 1. Introduction

Osteochondral defects, which include the lesions of both subchondral bone and articular cartilage, always occur attribute to various conditions such as trauma, aging, and inflammation. Previous study has indicated that full thickness chondral defects or osteochondral defects can lead to articular cartilage loss and the development of early osteoarthritis [1]. It is difficult to self-heal of articular cartilage after injury because of its unique characteristic of articular cartilage and the complex environment, such as confinement of resident chondrocytes to dense extracellular matrix (ECM) and the avascular property. Several surgical treatments have been proposed to repair such defects, such as microfracture, autograft, or allograft osteochondral transplantation and cell (stem cell or chondrocyte) transplantation [2–5]. Although these treatments have achieved good outcomes, some undesirable complications and failures are not uncommon [6–8]. So far, there is no consensus in regard to the most effective form of treatments. Therefore, new and effective therapies to articular cartilage lesions are needed urgently.

An innovative technology, termed cell sheet technology (CST), has been successfully used for tissue regeneration including heart, cornea, tooth repair, and so on [9–11]. CST can harvest seed cells without enzymatic digestion; therefore, abundant ECM was preserved. This technology is capable of harvesting seed cells with good vitality and maintaining a large amount of ECM. On the one hand, the ECM provides indispensable mechanical support and high adhesive ability (owing to fibronectin) for maintaining the integrality of the cell sheet during transplanting to the host tissues. On the other hand, the ECM provides the favorable microenvironment for cell growth by participating in adjusting various aspects of cell migration, proliferation, and differentiation [12, 13]. Currently, the patterns of CST have evolved from monolayer pure cell sheet to homogeneous or heterogeneous multilayer cell sheet. For example, Zhang et al. fabricated a double-cell sheet complex composed of a vascular endothelial cell sheet and an osteogenic cell sheet to explore the blood vessel formation potential and osteogenesis ability [14]. Fan et al. constructed the prevascularized nerveconduits based on the prevascularized stem cell sheet to assess their repair effects on spinal cord injury (SCI) of rats [15].

Various types of seed cells have been used to fabricate cell sheet, including bone marrow mesenchyml stem cells (BMSCs) [16], adipose tissue-derived stromal cells (ADSCs) [17], periodontal ligament-derived cells (PDLCs) [18–20], dentalfollicle cells (DFCs) [21], and gingival margin-derived cells (GMCs) [22]. In cartilage tissue engineering, many studies have used chondrocytes as seed cells to form chondrocyte sheet for the repair of cartilage defects [23, 24]. However, chondrocytes are apt to dedifferentiate during in vitro culture. When the dedifferentiated chondrocytes are transplanted in vivo, fibrocartilage will form in cartilage defects, which is inferior to normal cartilage that composes of hyaline cartilage [25]. Therefore, it is necessary and urgent to seek the ideal cell sources used to form cell sheet for cartilage regeneration. Human amniotic mesenchymal stem cells (hAMSCs) have attracted many researchers' attention because of their no ethical controversies, noninvasive and convenient collection, low immunogenicity, high viability, proliferation, and multilineage differentiation ability. What is more, hAMSCs are capable of chondrogenic differentiation under special laboratory conditions [26]. So far, hAMSCs have been widely applied in various fields and show promising prospect in cartilage regeneration [27, 28].

**Figure 1:**
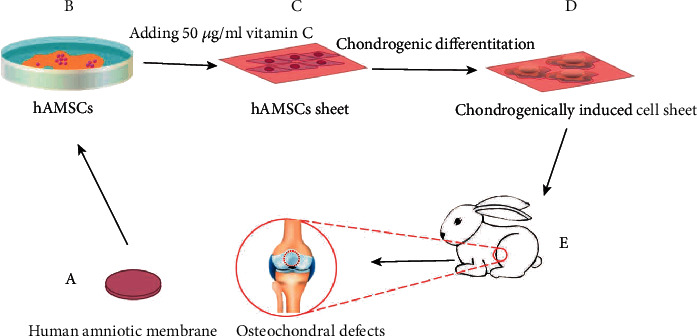
The schematic diagram of the experimental operation. (a) Harvesting human amniotic membrane from placentas. (b) Isolating hAMSCs from the human amniotic membrane of placentas by twice digestion with trypsin and once digestion with collagenase type II. (c) Fabricating hAMSC sheet by adding 50 *μ*g/ml vitamin C into cell sheet induction medium. (d) Fabricating chondrogenically induced hAMSC sheet by changing for cell sheet induction medium for chondrogenic induction medium. (e) Creating rabbit osteochondral defects in the center of the femoral trochlear groove and the osteochondral defects were treated with nothing, noninduced cell sheet and chondrogenically induced cell sheet. hAMSCs: human amniotic mesenchymal stem cells.

Although our previous study has demonstrated that hAMSC sheet could facilitate repair of rabbit osteochondral defects, no studies have compare the effect of noninduced hAMSCs sheet with chondrogenically induced hAMSC sheet on osteochondral defects. Therefore, this study is aimed at comparing the effect of noninduced hAMSC sheet with chondrogenically induced hAMSC sheet on osteochondral defects and cartilage regeneration. Our hypothesis for this study was that chondrogenically induced hAMSC sheet had the better cartilage repair effect compared with noninduced hAMSC sheet. The schematic diagram of the experimental operation is showed in Figure 1.

## 2. Materials and Methods

### 2.1. Isolation and Cultivation of hAMSCs

Placentas used in this experiment were all harvested from the Obstetrics Department of the Affiliated Hospital of Zunyi Medical University, and related informed consents were acquired from each donor before experiment. hAMSCs were isolated from the amnion of human placentas in accordance with a previous protocol [26]. Briefly, we first dissected bluntly human amniotic membrane from the placenta and then washed the amnion with aseptic phosphate-buffered saline (PBS) three times. Following this, the amnion was transferred to sterile containers at 4°C in a laboratory facility. The amnion was futher washed with PBS containing 1% penicillin and streptomycin (Solarbio, Beijing, China) and cut into approximate 1-2 mm^3^ pieces. Digestion was performed twice by addition of 0.125% trypsin (Solarbio, Beijing, China) for 30 minutes each and incubated for 40 minutes with 0.75% collagenase type II (Gibco, USA) at 37°C, until the pieces were indistinguishable. LG-DMEM/F12 medium containing 10% fetal bovine serum (FBS), 1% penicillin/streptomycin, 1% L-glutamine, 1% nonessential amino acids, and 5 ng/ml basic fibroblast growth factor (PeproTech) was used to culture the hAMSCs. hAMSCs were harvested and seeded in dishes at 37°C, with 5% humidified CO_2_. The medium was changed every 3 days, and hAMSCs were passaged when cells reached 80% confluence. Third generation (P3) hAMSCs were used for subsequent experiments.

### 2.2. Multidirectional Differentiation Potential of hAMSCs

P3 hAMSCs were seeded in six-well plates at the density of 1 × 10^5^ cells/cm^2^. When hAMSCs reached 60% confluence, cells were induced for osteogenic differentiation and chondrogenic differentiation, respectively. For osteogenic differentiation, hAMSCs were cultured with the osteogenic medium for 21 days. The osteogenic medium contained 10% fetal bovine serum (FBS), 1% penicillin/streptomycin, 1% L-glutamine, 1% nonessential amino acids, 50 *μ*g/ml vitamin C, 10 mM *β*-glycerophosphate, and 10 nM dexamethasone (Solarbio, Beijing, China). Afterwards, the osteogenesis was detected by alzarin red staining (Solarbio, Beijing, China). For chondrogenic differentiation, hAMSCs were cultured with chondrogenic differentiation medium (Chondrogenic-Inducer Reagent; Cyagen) for 14 days and assessed by alcian blue staining (Solarbio, Beijing, China).

### 2.3. Construction of hAMSC Sheet

P3 hAMSCs were cultured in six-well culture plates at a density of 1 × 10^6^ cells/cm^2^. After cultivation in normal medium for 48 h, cell sheet induction medium was added, which supplemented with 50 *μ*g/ml vitamin C, 10% (v/v) FBS, 1% (v/v) P/S, 1% (v/v) glutamine, and 1% (v/v) nonessential amino acids. The cell sheet induction medium was changed every two days, and hAMSCs were cultured for 7 days without passaging to obtain a dense and contiguous cell sheet. The hAMSC sheet was formed owing to the large amount of binding protein and fibronectin secretion, which were produced by the influence of induction medium. The hAMSC sheet was harvested by slightly detaching the cell sheet from the six-well culture plate using a cell scraper. Then, the characterization of hAMSC sheet was evaluated in subsequent experiments.

### 2.4. Characterization of hAMSC Sheet

hAMSC sheet was evaluated macroscopically and microscopically. Macroscopically, we observed the appearance and color of the hAMSC sheet. Microscopically, we observed the cellular morphology under inverted phase contrast microscope. To assess the viability of hAMSCs in the cell sheet, live/dead staining was carried out by a live/dead stain kit (Invitrogen, USA). After culture for cell sheet, hAMSCs were stained with live/dead working solution for 30 minutes at room temperature. Stained cells were photographed by automated fluorescence microscope. For scanning electron microscopy (SEM) analysis, hAMSC sheet was fixed with 3% glutaraldehyde in 0.1 M PBS for 2 h at 4°C, followed by fixing in 1% osmium tetroxide for 60 minutes at room temperature and serial dehydration with ethanol. Then, the samples were air dried, mounted, sputter coated with gold, and tested using the Hitachi SU8100 SEM (Hitachi, Tokyo, Japan). For histological assessment, the hAMSC sheet was fixed in 4% buffered paraformaldehyde, dehydrated in graded alcohols, embedded in paraffin, and cut into sections. Sections were stained with hematoxylin-eosin (HE) to observe tissue structure.

### 2.5. Chondrogenic Induction of hAMSC Sheet

P3 hAMSCs were seeded in six-well culture plates at a density of 1 × 10^6^ cells/cm^2^. First, hAMSCs were cultured in general medium for two days, and then the cell sheet induction medium was added to further continuously cultured for 7 days. Afterwards, the cell sheet induction medium was changed to chondrogenic induction medium, and cells were constantly cultured for another 14 days. Then, SEM and quantitative real-time reverse transcriptase-PCR (qRT-PCR) were used to detect chondrogenic differentiation of cell sheet.

### 2.6. Quantitative Real-Time Reverse Transcriptase-PCR (qRT-PCR)

The total RNA was extracted from the noninduced cell sheet and chondrogenically induced cell sheet by RNAiso plus (Takara Bio Inc, Shiga, Japan) according to manufacturer's instructions, and the cDNA was synthesized by a PrimeScript RT Reagent Kit (Takara Bio Inc, Shiga, Japan). Then, the expressions of SOX9 and collagen type II (COL-II) were detected by quantitative real-time PCR with TB Green Premix Ex Taq (Takara Bio Inc, Shiga, Japan). The gene expressions of SOX9 and COL-II were normalized to human glyceraldehyde 3-phosphate dehydrogenase (GAPDH) and calculated by using the 2^-*ΔΔ*Ct^ method. The primer sequences were shown in Table 1.

### 2.7. Surgical Procedure and Creation of Osteochondral Defects

**Figure 2:**
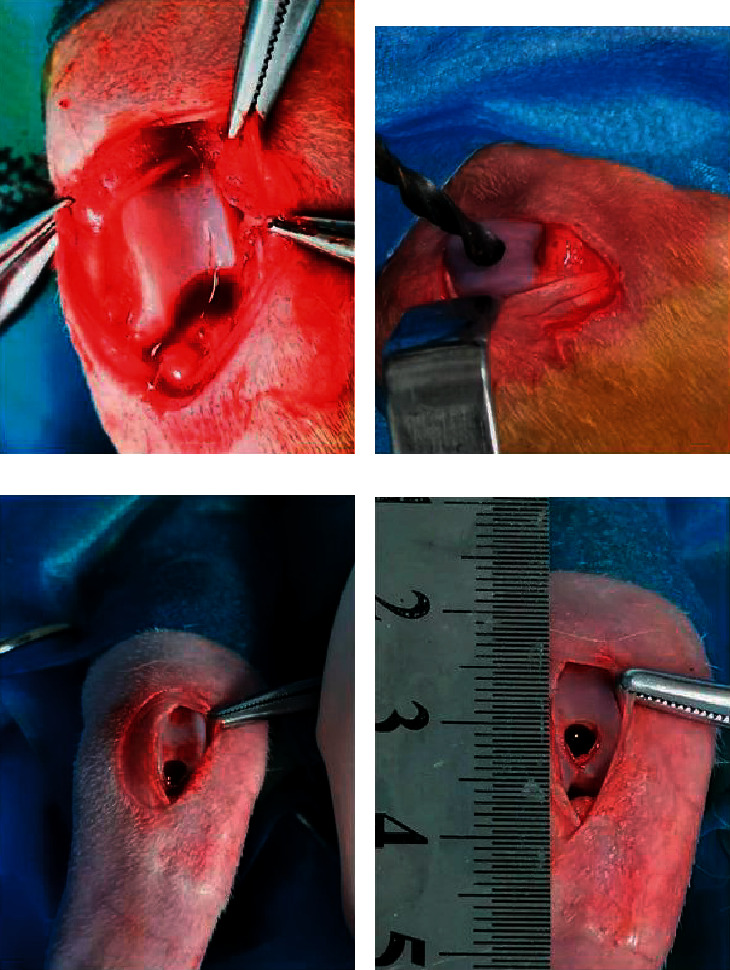
The operative process of creation osteochondral defects. (a) Exposing the articular surfaces of the trochlear grooves. (b)–(d) Creating osteochondral defects at the right knee joints (diameter: 3.5 mm; depth: 3 mm).

All of the New Zealand white rabbits utilized in this experiment were treated according to the guidelines approved by the Ethics Committee of Affiliated Hospital of Zunyi Medical University. The experiment was divided into 3 groups: group A, control group (defects were treated with nothing); group B, noninduced cell sheet group (defects were treated with non-induced cell sheet); and group C, chondrogenically induced cell sheet group (defects were treated with induced cell sheet). All procedures were performed as described in previous article. In brief, animals were anesthetized by intramuscular injection of 0.75 ml/kg of 3% sodium pentobarbital and were placed in the supine position. After shaving and sterilizing, the medial parapatellar incision was conducted at the right knee joint. Then, the patella was dislocated laterally to expose the articular surfaces, and osteochondral defects (diameter: 3.5 mm; depth: 3 mm) were made in the center of the femoral trochlear groove (Figure 2). 15 defects were created and then divided into 3 groups: control group (*n* = 5); noninduced cell sheet group (*n* = 5); and chondrogenically induced cell sheet group (*n* = 5). After surgery, penicillin was used to prevent infection. The animals were sacrificed at 12 weeks, and the distal femurs were harvested for subsequent assessments.

### 2.8. Macroscopic Evaluations

After sacrifice, each rabbit in the 3 groups was blindly assessed by 3 observers based on the ICRS scores [29]. The assessment was conducted from 3 dimensions: macroscopic appearance, degree of defect repair, and integration to border zone. The score classification was as follows: 1-3 points, severely abnormal; 4-7 points, abnormal; 8-11 points, basically normal; and 12 points, completely normal. Macroscopic images of the defects were taken for assessment.

### 2.9. Histological Examination

The specimens were fixed in 4% paraformaldehyde for 24 hours and decalcified for approximate 2 weeks. Then, they were embedded in paraffin for histological sectioning. Sagittal sections of about 5 *μ*m in thickness were harvested and stained with HE, toluidine Blue (TB), Safranin-O/Fast-green (SO/FG), and subjected to immunohistochemical staining of collagen type I (COL-I) and COL-II. Sections were randomly examined and scored in accordance with the O'Driscoll histological grading scale (Table 2) and the modified Mankin's histological score. The modified Mankin's histological score was scored on a scale of 0-6, where 0 = normal; 1 = irregular surface, including fissures into the radial layer; 2 = pannus; 3 = absence of superficial cartilage layers; 4 = slight disorganization (cellular row absent, some small superficial clusters); 5 = fissure into the calcified cartilage layer; and 6 = disorganization (chaotic structure, clusters, and osteoclasts activity). Cellular abnormalities were scored on a scale of 0-3, where 0 = normal; 1 = hypercellularity, including small superficial clusters; 2 = clusters; and 3 = hypocellularity. The matrix staining was scored on a scale of 0-4, where 0 = normal/slight reduction in staining; 1 = staining reduced in the radial layer; 2 = staining reduced in the interterritorial matrix; 3 = staining present only in the pericellular matrix; and 4 = staining absent.

### 2.10. Statistical Analysis

The data are expressed as the means ± standard deviations. Statistical analysis (GraphPad Prism 7.0 software, USA) was performed by one-way ANOVA followed by Tukey's multiple comparison test for further evaluation of the differences between the groups unless otherwise stated. *P* < .05 was considered to indicate statistical significance.

## 3. Results

### 3.1. Isolation, Cultivation, and Multidirectional Differentiation Potential of hAMSCs

**Figure 3:**
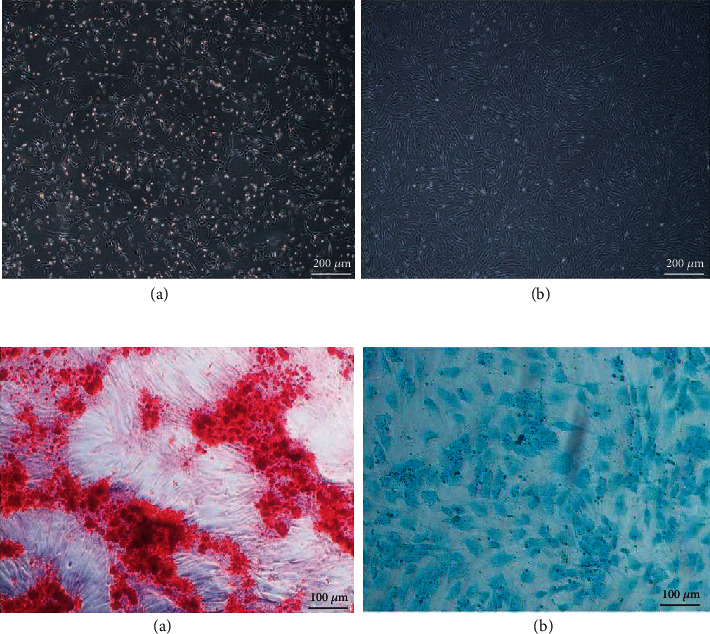
(a) Microscopic observations of hAMSCs. (A) P0 hAMSCs. (B) P3 hAMSCs. Scale bar: 200 *μ*m. (b) Multidirectional differentiation potential for osteogenic and chondrogenic differentiation of passage 3 hAMSCs. (A) Osteogenic differentiation (alzarin red staining). (B) Chondrogenic differentiation (alcian blue staining). Scale bar: 100 *μ*m.

The morphology of cultured hAMSCs was observed under inverted phase contrast microscope. Primary and P1, P2, and P3 hAMSCs all showed the monolayer of adherent cells and demonstrated the spindle-shaped exterior with radial-like growth, and increasing polarization was observed with each passage. P3 hAMSCs exhibited a uniformly vortex-likeshape (Figure 3(a)). During the isolation process of hAMSCs, small portions of human amniotic epithelial cells from amnion were present in hAMSCs. To reduce the rate of epithelial cells in hAMSCs, we passaged cells and obtained highly pure hAMSCs. To verify the stemness of hAMSCs, multidirectional differentiation potential was detected. The results of alzarin red staining and alcian blue staining indicated that hAMSCs possessed the potential for multidirectional differentiation into osteoblasts and chondrocytes (Figure 3(b)).

### 3.2. Construction and Characterization of hAMSC Sheet

**Figure 4:**
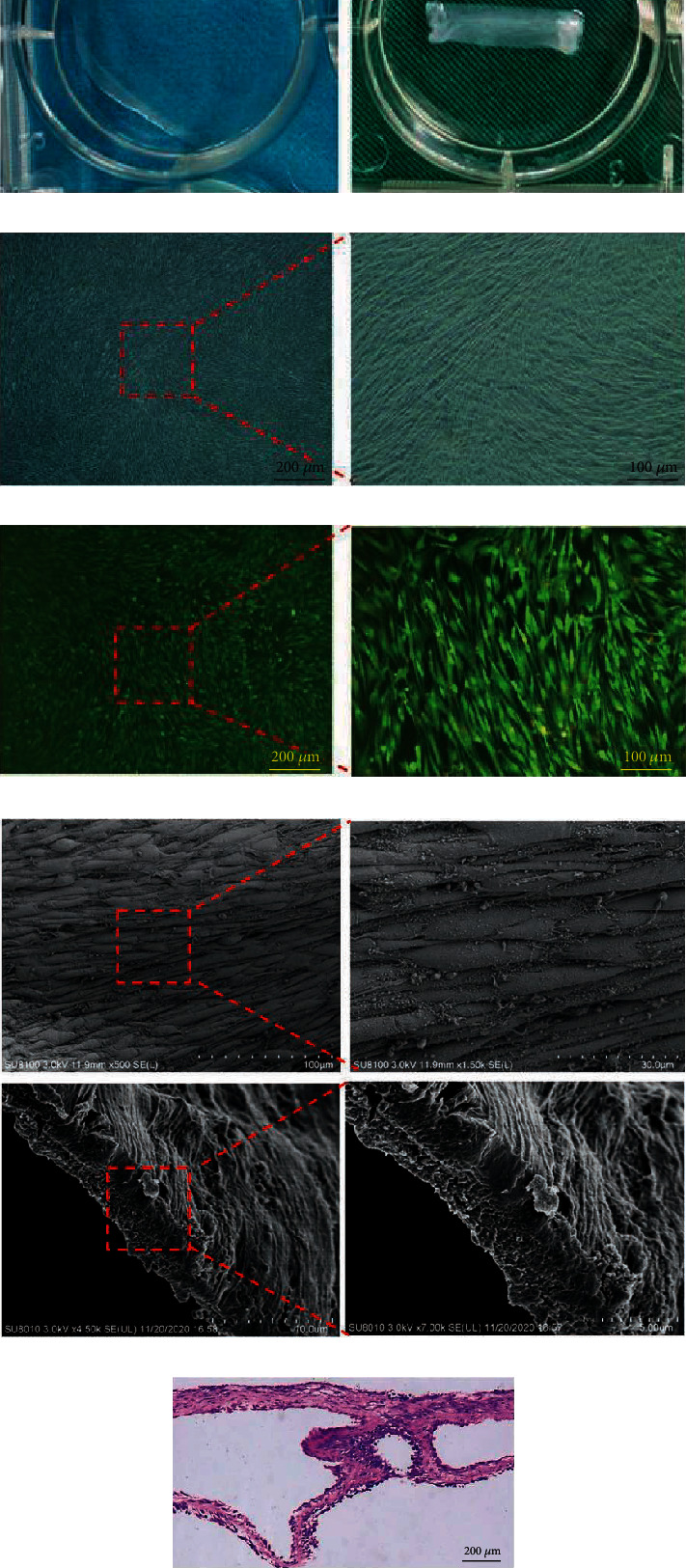
Preparation and characterization of hAMSCs sheet. (a) Gross appearance of the hAMSC sheet derived from hAMSCs and the cell sheet could be folded to an oblong-shaped flap. (b) hAMSC sheet was observed under inverted phase contrast microscope. Many long spindle-shaped cells were closely arranged and evenly distributed. (c) Live/dead staining of the cell sheet. Green indicated live cells and red indicated dead cells. (d) SEM of cell sheet. Cells were embedded in a large amount of extracellular matrix secreted by themselves. (e) Histological analysis (HE staining) of the harvested cell sheet that was composed of multilayer cells and vast ECM.

After 7 days of culture in cell sheet medium, a uniformly white cell sheet was formed in the six-well culture plate. The high-density hAMSCs proliferated rapidly and formed a thick cell sheet with continuous cultivation. The formed hAMSC sheet could be separated from the culture plate by a cell scraper and could be fabricated easily into special shape (Figure 4(a)). Under the inverted phase contrast microscope, many long spindle-shaped cells were closely arranged and evenly distributed. In addition, vast ECM was produced by hAMSCs (Figure 4(b)). To test the viability of hAMSCs in the cell sheet, live/dead staining was used, the results showed the cells were all well-lived, and most of them were live cells (green fluorescence) with good morphology and few dead cells (red fluorescence, Figure 4(c)). The results of SEM indicated that hAMSC sheet secreted a large amount of ECM, and cells were embedded in the ECM (Figure 4(d)). The result of HE staining showed that hAMSC sheet was composed of multilayers of hAMSCs that secreted a large amount of ECM (Figure 4(e)).

### 3.3. Chondrogenic Induction of Cell Sheet

**Figure 5:**
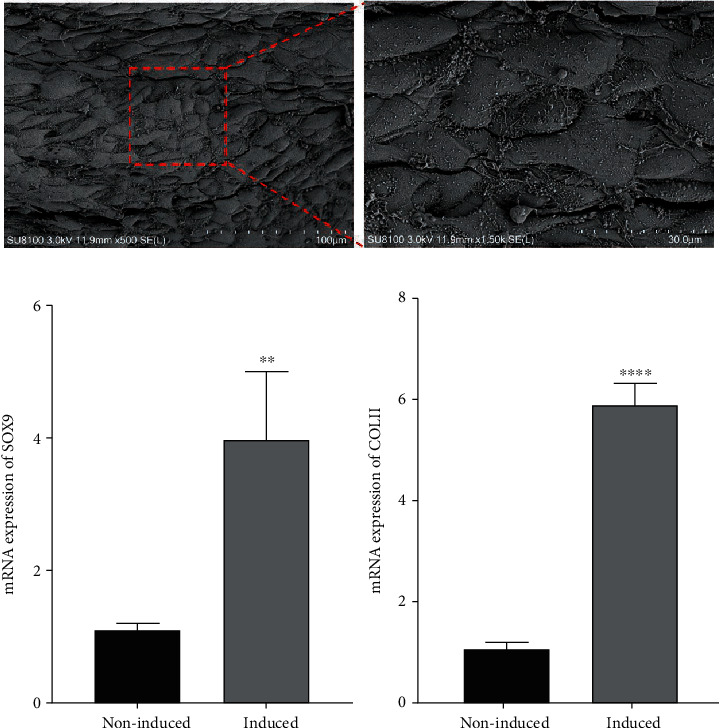
Chondrogenic induction of hAMSC sheet. (a) The SEM results of hAMSCs sheet induced by chondrogenic induction medium. (b) mRNA level of SOX9 and collagen type II. Compared with the noninduced group, mRNA expressions of SOX9 and collagen type II were significantly increased in the induced group on day 14 (^∗∗∗∗^*P* < .0001).

After induction in chondrogenic induction medium, the white membranous substance was produced at the bottom of the six-well plates. The cellular morphology was changed after induction. SEM results showed that the round or elliptical cells were closely arranged and evenly distributed, and a large amount of ECM was around cells (Figure 5(a)). The gene expressions of SOX9 and COL-II were analyzed by qRT-PCR after chondrogenic induction for 14 days. The expressions of SOX9 and COL-II were increased in the chondrogenically induced group as compared with the noninduced cell sheet group (Figure 5(b)).

### 3.4. Gross Observation

**Figure 6:**
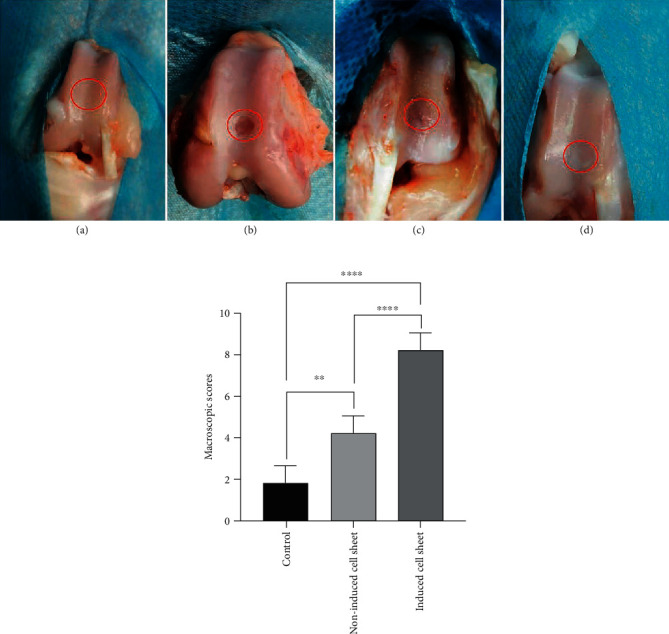
(a) Macroscopic observations of regenerative cartilage at 12 weeks after surgery. (A) Normal articular cartilage. (B) Control group. (C) Noninduced cell sheet group. (D) Chondrogenically induced cell sheet group. (b) International Cartilage Repair Society macroscopic scores of all groups at 12 weeks after surgery. ^∗∗^*P* < .01; ^∗∗∗∗^*P* < .0001.

During the observational period, no wound/joint infection and deaths occurred in all groups, and all samples were followed. At 12 weeks after surgery, regenerative tissue in the chondrogenically induced cell sheet group had filled the defects entirely, and the surface of the regenerative cartilage was smooth, which was similar to native cartilage. The color of the regenerated cartilage was also similar to native cartilage. In addition, there was no obvious boundary between the native cartilage and regenerated tissue. In the noninduced cell sheet group, although the regenerative tissue partially filled osteochondral defects and the boundary between the native cartilage and regenerated tissue was partially distinguished, the irregular and coarse surface of regenerative cartilage was identified. In the control group, we could observe the distinct cavity that was filled with a small amount of newly formed tissue. The boundary could be easily distinguished, and the color and the surface of newly formed tissue were different from the native cartilage (Figure 6(a)). The ICRS macroscopic scores were higher in the chondrogenically induced cell sheet group than the noninduced cell sheet group and the control group. Besides, the scores of the noninduced cell sheet group were higher than that of the control group (Figure 6(b)).

### 3.5. Histological Examination

**Figure 7:**
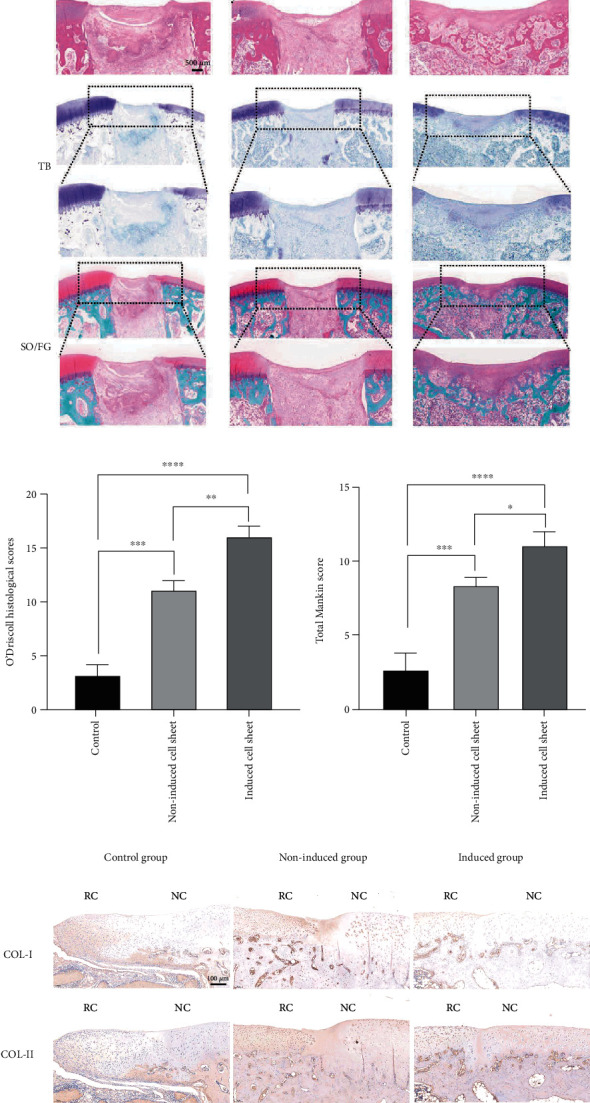
(a) Histological examination of regenerated tissue in all groups at 12 weeks postoperatively. TB: toluidine blue; SO/FG: Safranin-O/Fast-green. Scale bar: 1 mm and 500 *μ*m. (b) O'Driscoll histological scores of groups at 12 weeks postoperatively, ^∗∗^*P* < .01, ^∗∗∗^*P* < .001, ^∗∗∗∗^*P* < .0001. (c) The Mankin scores of groups at 12 weeks postoperatively, ^∗^*P* < .05, ^∗∗∗^*P* < .001, ^∗∗∗∗^*P* < .0001. (d) Immunohistochemical staining of COL-I and COL-II. Scale bar: 100 *μ*m. RC: regenerated cartilage; NC: normal cartilage.

Histological staining assessment further confirmed that the chondrogenically induced cell sheet group had the better repair effect than the noninduced cell sheet group and the control group. At 12 weeks, HE staining result of the control group showed there was almost no newly formed cartilage, and only some irregular tissues filled in the defects. The boundary between the adjacent normal cartilage and regenerative cartilage was obvious, and the defects center still remained vacant. Besides, the regenerative tissues were negative for SO/FG and TB staining. In the noninduced cell sheet group, we could observe some newly regenerative tissues filling the defects. The continuity and thickness of the regenerative tissues were worse than those of the induced cell sheet group. As observed by SO/FG staining and TB staining, the results showed that glycosaminoglycans (GAGs) were less than those of the induced cell sheet group. In the induced cell sheet group, we could not only observe newly formed cartilage but also find the continuous and smooth cartilage that was similar to the native cartilage. The results of SO/FG and TB were all superior to other groups, which demonstrated that mass GAGs were formed. The O'Driscoll histological repair scores were higher in the induced cell sheet group than in other groups (*P* < .05). The scores of the noninduced cell sheet group were higher than that of the control group (*P* < .05). The scores of the control group were the lowest (Figure 7(b)). Similarly, the Mankin scores were higher in the induced cell sheet group than the other groups (*P* < .05). The scores of the noninduced cell sheet group were higher than that of the control group (*P* < .05). The scores of the control group were the lowest (Figure 7(c)). The expression of COL I and COL II was qualitatively observed by immunohistochemical staining. The results of COL-II were strongly positive in the induced cell sheet group than the other groups. Since COL-II mainly exists in hyaline cartilage, the positive rate of COL-II immunohistochemistry in the induced cell sheet group demonstrated that hyaline cartilage formed. However, the COL-I immunohistochemical staining in the induced cell sheet group showed negative staining while the results were positive in the other groups, which indicated that fibrous cartilage formed in the control group and the non-induced cell sheet group. Besides, the continuity and smoothness of the newly formed cartilage in the control group and the noninduced cell sheet group were inferior to those of the induced cell sheet group and the normal cartilage.

## 4. Discussion

This study has demonstrated that noninduced cell sheet and chondrogenically induced cell sheet could both repair osteochondral defects. We fabricated hAMSC sheet by a simple and effective method (adding 50 *μ*g/ml vitamin C) to stimulate hAMSCs to secrete a large amount of ECM. The hAMSC sheet had differentiative potential into chondrocytes, and we further induced the hAMSC sheet to prepare chondrogenically induced cell sheet. In vivo, we demonstrated that noninduced cell sheet and chondrogenically induced cell sheet could both repair osteochondral defects, and the chondrogenically induced cell sheet had the better repair effect than the noninduced cell sheet. Although the potential mechanisms of this treatment were not definite, hAMSC sheet played the crucial role in osteochondral repair and cartilage regeneration, especially the chondrogenically induced hAMSCs sheet.

Osteochondral lesion is the clinically common disease, which is caused by serious traumas, infections, or degenerative diseases of joints. If the lesions are not treated timely and effectively, these cases will eventually develop into osteoarthritis accompaning by joint deformity and functional disorders [30, 31]. The regenerative capacity of articular cartilage is very low owing to the avascular trait and the limited nutrient supply in the surrounding synovial fluid [32]. It is the great challenge to reconstruct osteochondral regions for every clinician. Many approaches have been used to treat osteochondral lesions including microfracture, arthroscopic debridement, cell-based approaches, and autologous chondrocyte implantation (ACI). However, every treatment had its own drawback, such as donor morbidity, the limited source of cells, and possible immune rejection. The reports have demonstrated that bioactive cues derived from ECM can recruit vast endogenous stem cells to injury sites and guide the self-healing progress of lesions [33, 34]. Besides, the ECM plays an important role in the proliferation and differentiation of cells surrounding articular cartilage because of the dynamic interactions between the ECM and these surrounding cells. In addition, the ECM around cartilage is capable of providing nutrients and mechanical support for chondrocytes [35]. Therefore, the ECM aroud articular cartilage plays an important role in cartilage regeneration.

CST has arisen as a reliable alternative to harvest seed cells and preserve abundant ECM, which was developed in the 1990s and is now applied in tissue engineering mostly as scaffold-free construction. Owing to its unique advantages, CST has been used in various fields. The foremost benefit of CST is the absence of the enzymatic digestion step before immobilization on the scaffold or implantation in vivo. Therefore, cell surface proteins [36], intercellular junctions, and the connections of cells with surrounding ECM are preserved, which provide mechanical support and suitable microenvironment for the cells. What is more, the adundant ECM has the significant effect on cell migration [37], proliferation, and differentiation and avoids accelerating cellular changes in phenotype, downregulation of cell growth, cellular senescence, and the loss of multipotency of stem cells [38]. There are several methods to fabricate cell sheet, including the temperature-responsive culture dish method, light reaction, electroreaction, and pH systems. Nevertheless, all of these methods have some defects. For instance, it is difficult to detach cell sheet in the light reaction system [39]; the cells in the pH system are easily damaged [40]; cells growth is suppressed by the coated materials in the electroreaction system [41]; cell aggregation and differentiation can be affected by the temperature-sensitive polymer [42]. In this study, we fabricated cell sheet by adding 50 *μ*g/ml vitamin C into cell sheet induction medium to produce a large amount of ECM, and then the formed cell sheet was detached from the culture plates by mechanical method. This method to induce the formation of cell sheet was simple and efficient, which was accepted by many researchers and has been widely used in some experiments. What is more, in our previous study, we have successfully constructed cell sheet by this method [43].

At present, various types of cells have been used to fabricate cell sheet, including epithelial cells [44], stem cells [45], and pluripotent stemderived cells [46]. For cartilage regeneration, the most type of cells applied to construct cell sheet was chondrocyte. However, the inherent disadvantage of chondrocyte is the dedifferentiaton, which prevents the wide application of chondrocyte sheet in fundamental researches and clinical practice. hAMSCs, the newly discovered MSCs, have many advantages compare with other MSCs [47]. First, hAMSCs have the expansive sources, which are abundantly available from placentas after birth. Second, there are no ethical or moral limitations, and the extraction process of hAMSC causes no trauma. What is more, hAMSCs express low levels of costimulatory molecules (CD80, CD83, and CD86) and major histocompatibility type I antigens (HLA-A, HLA-B, and HLA-C), which demonstrates that hAMSCs have low immunogenicity and have no immunological rejection to host in transplantation [48]. Therefore, hAMSCs have been used as seed cells to fabricate cell sheet for repair osteochondral defects in this study. In cartilage tissue engineering, BMSCs are also widely used to fabricate cell sheet for cartilage regeneration. Thorp et al. used hBMSCs to fabricate scafold-free, hyaline-like cartilage articular cartilage regeneration [49]. Qi et al. demonstrated the novel method of incorporation of rabbits BMSC sheet to PLGA/MCSs could enhance the ability of cartilage regeneration and integration between repair cartilage and the surrounding cartilage [50]. As compared with other MSC-derived cell sheet (such as BMSC sheet), the hAMSC sheet have some advantages, including the expansive sources, no ethical or moral limitations, and low immunogenicity. However, there is still no research for comparison the effect of hAMSC sheet with other MSC-derived cell sheet on caitilage regeneration. Therefore, we will perform further study to compare the cartilage repair effect of hAMSC sheet with other MSC-derived cell sheet.

In vitro, we successfully constructed hAMSC sheet by adding 50 *μ*g/ml vitamin C into cell sheet induction medium and demonstrated that hAMSC sheet possessed a multilayered structure, and the cells were evenly distributed and closely arranged. Besides, we further induced the hAMSC sheet to prepare chondrogenically induced cell sheet. The chondrogenically induced cell sheet was prepared, and the expressions of SOX9 and Col-II increased compared with noninduced cell sheet.

In vivo, macroscopic and histological assessments demonstrated that both noninduced cell sheet and chondrogenically induced cell sheet promoted the cartilage repair, while the chondrogenically induced cell sheet had the better cartilage repair effect than noninduced cell sheet. The following possible reasons might explain the above results. First, the constructed hAMSC sheet did not need enzymatic digestion, retaining a large amount of ECM and many cytokines, such as fibroblast growth factor, colony stimulating factor, and transforming growth factor-*β*. The ECM and abundant cytokines had the positive effect on cell survival, migration, and differentiation [51]. Second, the implanted hAMSCs had paracrine effects, which might attract progenitor cells to participate in the cartilage regeneration process [48]. The implanted hAMSC sheet might had positive effect on recruit the cells surrounding the osteochondral defects, such as synovial mesenchymal stem cells in knee joint, and BMSCs locating under subchondral bone. Third, the barrier and adhesion function of implanted hAMSC sheet could protect injured cartilage from catabolic cytokines in knee joint fluid [52]. Therefore, proteoglycan loss could be avoided, and regenerated cartilage degeneration was reduced. Previous studies demonstrated that hydrogels had promising applications in delivery of seed cells or drugs to enhances in situ tissue regeneration, such as osteochondral and bone regeneration, which provided new direction for our future researches [53–55]. Therefore, we will focus on exploring the effect of combining hydrogel (such as host-guest macromer hydrogel) and cell sheet on osteochondral regeneration. For instance, we can combine cell sheet and hydrogel fibrous scaffolds that are fabricated by electrospinning technique to generate composite scaffolds and explore their effect on osteochondral regeneration or other tissue regeneration.

This study also had some limitations. First, we did not track the transplanted hAMSC sheet in vivo after surgery. Therefore, it was unknown whether hAMSC sheet could survive in osteochondral defects, and the concrete mechanism of osteochondral repair was not explicit. Further studies are needed to explore the fate of hAMSC sheet after transplantation into osteochondral defects. Second, we only set one time point to evaluate the cartilage repair in this study. The cartilage healing is a gradually developing process; so, different time points should be chosen to explore the healing process in further studies. Third, the rabbit model was used in this study, which was different from the clinical cases. This small animal model has its limitation: the depth of the cartilage defects could not be controlled well. So, further large animal researches are required to explore the effect of hAMSC sheet on osteochondral regeneration. Last, mechanical test was not performed to assess the regenerated cartilage in this study. Therefore, further biomechanical evaluations of regenerated cartilage are necessary to fully evaluate the biomechanical properties of repaired tissue.

## 5. Conclusions

This study has demonstrated that noninduced cell sheet and chondrogenically induced cell sheet could both repair osteochondral defects, and the chondrogenically induced cell sheet had the better repair effect than the noninduced cell sheet. hAMSCs can be the feasible seed cells to prepare cell sheet. To clarify the underlying mechanism of osteochondral repair, further studies are required.

## Figures and Tables

**Table 1:** Primer sequences for quantitative RT-PCR analysis.

Gene name	Gene symbol	Primer sequence	Accession number
Sex determining region	SOX9	F:TAAAGGCAACTCGTACCCAA	NM_000346.3
Y-box 9		R:ATTCTCCATCATCCTCCACG	
Collagen type II	COLII	F:CCTCTGCGACGACATAATCT	NM_001844.4
		R:CTCCTTTCTGTCCCTTTGGT	
Glyceraldehyde-3-phosphate dehydrogenase			NM_002046.4
	GAPDH	F:GCCTTCCGTGTCCCCACTGC	
		R:CAATGCCAGCCCCAGCGTCA	

**Table 2:** O'Driscoll histological cartilage repair score.

Characteristics	Score
Nature of predominant tissue	
Cellular morphology	
Hyaline articular cartilage	4
Incompletely differentiated mesenchymal	2
Fibrous tissue or bone	0
Safranin O staining of the matrix	
Normal or nearly normal	3
Moderate	2
Slight	1
None	0
Structure characteristics	
Surface regularity	
Smooth and intact	3
Superficial horizontal lamination	2
Fissures 25% to 100% of the thickness	1
Severe disruption including fibrillation	0
Structural integrity	
Normal	2
Slight disruption including cysts	1
Severe disintegration	0
Thickness	
100% of normal adjacent cartilage	2
50% to 100% of normal cartilage	1
0% to 50% of normal cartilage	0
Bonding to the adjacent cartilage	
Bonded at both ends of graft	2
Bonded at one end or partially at both ends	1
Not bonded	0
Freedom from cellular changes of degeneration	
Hypocellularity	
Normal cellularity	3
Slight hypocellularity	2
Moderate hypocellularity	1
Severe hypocellularity	0
Chondrocyte clustering	
No clusters	2
<25% of the cells	1
25% to 100 of the cells	0
Freedom from degenerative changes in adjacent cartilage	
Normal cellularity, no clusters, normal staining	3
Normal cellularity, mild clusters, slight staining	2
Mild or moderate hypocellularity, slight staining	1
Severe hypocellularity, poor or no staining	0
Total	24

## Data Availability

The data used to support the findings of this study are available from the corresponding author upon request.
